# Decline in HIV Prevalence among Young Men in the General Population of Cotonou, Benin, 1998–2008

**DOI:** 10.1371/journal.pone.0043818

**Published:** 2012-08-28

**Authors:** Luc Béhanzin, Souleymane Diabaté, Isaac Minani, Catherine M. Lowndes, Marie-Claude Boily, Annie-Claude Labbé, Séverin Anagonou, Djimon Marcel Zannou, Anne Buvé, Michel Alary

**Affiliations:** 1 Unite de recherche en santé des populations (URESP), Centre de recherche FRSQ du CHA universitaire de Québec, Québec Canada; 2 Département de médecine sociale et préventive, Université Laval, Québec, Canada; 3 Dispensaire IST, Cotonou, Bénin; 4 Health Protection Agency, London, United Kingdom; 5 Department of Infectious Disease Epidemiology, Imperial College, London, United Kingdom; 6 Département de microbiologie, Hôpital Maisonneuve-Rosemont, Montréal, Canada; 7 Faculté des sciences de la santé, Université d’Abomey-Calavi, Cotonou, Bénin; 8 Centre national hospitalier universitaire, Cotonou, Bénin; 9 Unit of Epidemiology and Control of HIV/STD, Institute of Tropical Medicine, Antwerp, Belgium; 10 Institut national de santé publique du Québec, Québec, Canada; Yale School of Public Health, United States of America

## Abstract

**Objective:**

To assess changes in the prevalence of HIV and other sexually transmitted infections, as well as in different proximal and distal factors related to HIV infection, in the general population of Cotonou between 1998 and 2008, while an intensive preventive intervention targeting the sex work milieu was ongoing.

**Methods:**

A two-stage cluster sampling procedure was used to select the participants in each study. Subjects aged 15–49 who agreed to participate were interviewed and tested for HIV, syphilis, HSV-2, gonorrhoea and chlamydia. We used the Roa-Scott Chi-square test (proportions) and the Student’s *t* test (means) for bivariate comparisons, and adjusted logistic regression models taking into account the cluster effect for multivariate analyses.

**Results:**

HIV prevalence decreased significantly in men (3.4% in 1998 versus 2.0% in 2008, p = 0.048), especially in those aged 15–29 (3.0% to 0.5%, p = 0.002). Among men, the prevalence of gonorrhoea decreased significantly (1.1% to 0.3%, p = 0.046) while HSV-2 prevalence increased from 12.0% to 18.1% (p = 0.0003). The proportion of men who reported condom use at least once (29.3% to 61.0%, p<0.0001) and of those having attained a secondary educational level or more (17.1% to 61.3%, p<0.0001) also increased significantly. There was an overall decrease in the prevalence of syphilis (1.5% to 0.6%, p = 0.0003).

**Conclusion:**

This is the first population-based study reporting a significant decline in HIV prevalence among young men in an African setting where overall prevalence has never reached 5%. The decline occurred while preventive interventions targeting the sex work milieu were ongoing and the educational level was increasing.

## Introduction

Sub-Saharan Africa is still severely affected by the HIV epidemic. While accounting for only 12% of the world population, 68% of the 34 million people infected with HIV worldwide live in this region [1]. A recent analysis mainly using data from antenatal care attendees provided some evidence of a significant decline in HIV prevalence between 2000 and 2010 in several African countries [1]. Only a few population-based studies documented a decline in HIV prevalence in the general population of some African countries. Such a decline was mainly observed among young people in Eastern and Southern Africa countries with highly generalized epidemics. In Eastern Zimbabwe between 1998 and 2003, HIV prevalence significantly decreased by 23% (10.6% to 8.1%) among more educated men aged 17–29 years [2]. Between 2000 and 2008, in Botswana, South Africa and the United Republic of Tanzania, national population-based surveys showed a significant decline of 25% or more in HIV prevalence among young men with concomitant changes in HIV related risky behaviour [3]. In West Africa, no decline in HIV prevalence among young people was documented through population-based surveys. However, in the general population of Côte d’Ivoire, a decline in HIV prevalence was observed between 1989 (7.0%) [4] and 2005 (4.7%) [5] in adults (15–45 years of age) living outside Abidjan. This observation was however made using the data of two entirely independent studies not using the same methodology and without the same population coverage. In Mali and Niger, two Demographic and Health Surveys (DHS) conducted in 2001 and 2006 have shown low (between 0.4 and 0.9%) and stable HIV prevalence among young men [6]–[9]. In Benin, HIV prevalence in the adult general population was 1.2% in 2006 [10]. In this country as elsewhere in sub-Saharan Africa, HIV transmission is predominantly heterosexual [11] and, as in most West African countries, the epidemic is concentrated among female sex workers (FSWs) and their clients [12]. Specific preventive interventions targeting both groups have the potential to substantially reduce HIV transmission over time, first among themselves and then among the general population [13]–[16]. Accordingly, in 1992 the Canadian International Development Agency (CIDA) funded an intervention for FSWs involving fully integrated field outreach activities (including behavioural change communication, improved condom accessibility and promotion of correct condom use), routine check-ups and free treatment of sexually transmitted infections (STI) at a clinic dedicated to FSWs. The intervention was implemented in Cotonou, the largest city and economic capital of Benin. It was extended to six other cities and to the clients of FSWs from year 2000. The interventions with clients consisted in outreach activities coupled with referral to free and confidential STI clinics for men. Overall the intervention was followed by declines in HIV and STI prevalence among the FSWs and their clients [13], [15]. Since data from population-based HIV seroprevalence surveys are more accurate in estimating HIV prevalence in the general population than data from antenatal clinics (ANC) [17] and as part of a broader project aiming at evaluating the overall impact of FSW preventive interventions, we conducted a cross-sectional study of HIV/STI prevalence and sexual behaviour in the general population of Cotonou in 2008. The aim of the present study was to compare data on HIV/STI prevalence from the latter study with data collected in a previous cross-sectional study carried out in 1998 [18], in order to assess the change in the prevalence of HIV and other STI, as well as in different proximal and distal factors related to HIV infection, in the general population of Cotonou.

**Figure 1:**
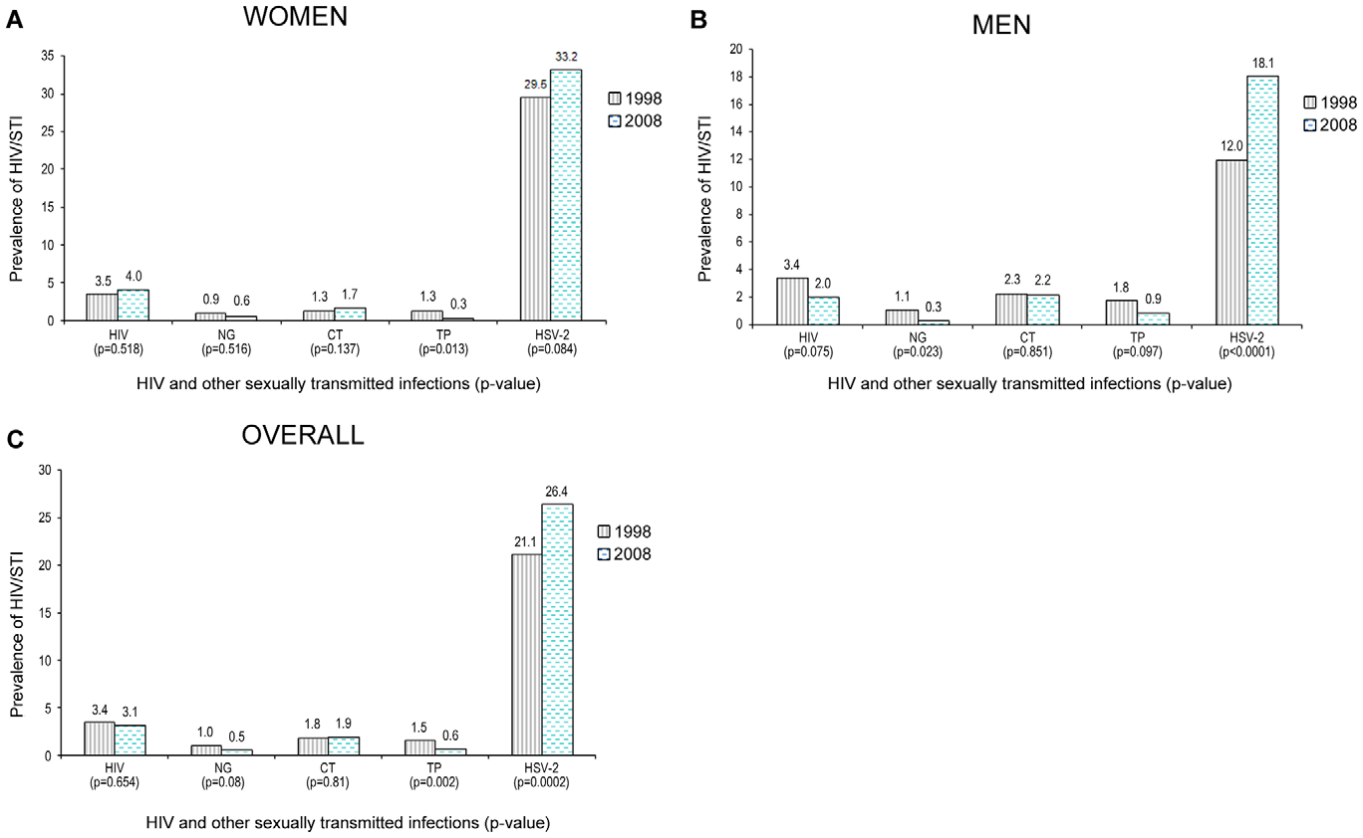
Univariate comparison of HIV/STI prevalence between 1998 and 2008 among women, Men and Overall, aged 15–49 years, general population of Cotonou, Benin. P-values are from Roa-Scott chi-square tests (chi-square tests adjusted for the cluster effect). NG, *Neisseria gonorrhoeae;* CT, *Chlamydia trachomatis*; TP, *Treponema pallidum;* HSV-2, Herpes simplex virus type-2.

**Table 1:** Multivariate comparison of HIV/STI prevalence between 1998 and 2008 among women and men aged 15–49 years, general population of Cotonou, Benin.

	Women^1^				Men^1^				Overall^1^			
	1998 (N = 1093)	2008 (N = 1348)	AOR (95%CI)	p-value	1998 (N = 1019)	2008 (N = 1159)	AOR (95%CI)	p-value	1998 (N = 2112)	2008 (N = 2507)	AOR† (95%CI)	p-value
HIV	35/1013 (3.5)	50/1239 (4.0)	1.13 (0.69–1.85)	0.638	31/926 (3.4)	21/1049 (2.0)	0.54 (0.29–0.99)	0.048	66/1939 (3.4)	71/2288 (3.1)	0.85 (0.56–1.30)	0.452
NG	9/974 (0.9)	8/1241 (0.6)	0.72 (0.24–2.16)	0.553	10/895 (1.1)	3/1040 (0.3)	0.26 (0.07–0.97)	0.046	19/1869 (1.0)	11/2281 (0.5)	0.48 (0.20–1.17)	0.108
CT	13/974 (1.3)	21/1230 (1.7)	1.28 (0.64–2.56)	0.477	21/895 (2.3)	23/1040 (2.2)	0.98 (0.51–1.87)	0.949	34/1869 (1.8)	44/2270 (1.9)	1.10 (0.65–1.87)	0.726
TP	12/960 (1.3)	4/1202 (0.3)	0.25 (0.08–0.80)	0.019	16/899 (1.8)	9/1007 (0.9)	0.47 (0.20–1.12)	0.089	28/1859 (1.5)	13/2209 (0.6)	0.37 (0.19–0.72)	0.003
HSV-2	275/932 (29.5)	397/1195 (33.2)	1.13 (0.91–1.40)	0.273	103/861 (12.0)	181/998 (18.1)	1.57 (1.23–1.99)	0.0003	378/1793 (21.1)	578/2193 (26.4)	1.26 (1.06–1.50)	0.008
TV	30/939 (3.2)	31/1221 (2.5)	0.84 (0.51–1.39)	0.511	–	–	–	–				

1 For women, men and overall figures, the first two columns of data are the prevalence figures in 1998 and 2008 respectively. *NG, Neisseria gonorrhoeae;*

CT, *Chlamydia trachomatis*;

TP, *Treponema pallidum;*

HSV-2, Herpes simplex virus-2;

TV, *Trichomonas vaginalis*;

CI, confidence interval;

AOR, adjusted odds ratio: adjusted for marital status and age (year of survey as a categorical variable (one dummy variable) with 1998 being the reference year);

† also adjusted for gender.

## Methods

### Sampling, Recruitment and Data Collection

During the studies conducted in 1998 and 2008, there were approximately 650,000 and 808,000 inhabitants in Cotonou, respectively [19]. A two-stage cluster sampling procedure was used to select the participants in both studies: the census areas were first selected using a probability proportional to size method and fixed number of households were then randomly selected from each census area [18]. In 1998 and 2008, respectively 950 and 1,070 households were sampled from 38 census areas. The selected households were visited and all the residents (women and men) aged 15–49 years were asked to participate in the studies. After being pre-tested and modified accordingly, structured, gender-specific questionnaires previously validated by the Joint United Nations Programme on HIV/AIDS (UNAIDS) were used to collect information on socio-demographic characteristics, condom use and sexual behaviour [20]. All the participants were asked to provide a blood sample to test for HIV, syphilis and herpes simplex virus type 2 (HSV-2) antibodies. A urine sample was collected to test for *Neisseria gonorrhoeae* (NG) and *Chlamydia trachomatis* (CT). A vaginal swab for *Trichomonas vaginalis* (TV) diagnosis was obtained from women. In 2008, dried blood spot (DBS) samples for HIV testing were obtained from participants who refused venipuncture, but who agreed to provide capillary blood. In participants who refused any blood collection, the urine sample was used for HIV testing, if consent was given for this procedure. The collection of specimens and the interviews were carried out in the participants’ houses.

### Laboratory Procedures

In 1998, HIV was tested with an enzyme-linked immunoassay (EIA; ICE HIV1/2; Murex Diagnostics, Dartford, UK) and confirmed with a rapid test (Capillus HIV1/2; Cambridge Diagnostics, Galway, Ireland). In 2008, two EIAs were used: Vironostika HIV1/2 (Biomérieux, Nancy, France) confirmed with Genie II® HIV1/2 (Bio-Rad, Marnes-la-Coquette, France) for both the serum and DBS samples. In 2008, for participants who provided only urine samples for HIV testing, the first test was an EIA (Calypte Biomedical Corporation, Berkeley, California, USA), followed by Western Blot (Calypte Biomedical Corporation’s Cambridge Biotech Co., Worcester, Mass) confirmation. Quality control for HIV testing was carried out on all the positive tests and 2% of the negative ones [21]. For HSV-2, serum samples were tested with two different HSV-2 Type Specific IgG ELISAs (Gull Laboratories, Bad Homburg, Germany in 1998 and Kalon Biological, Surrey, United Kingdom in 2008), since the Gull test (used in 1998) was withdrawn from the market before 2001 [22]. Syphilis diagnosis on serum samples was based on the same tests in both rounds: a positive rapid plasma reagin (RPR) Card Antigen Suspension (Becton Dickinson & Co., Cockeysville, Maryland, USA) confirmed by a positive *Treponema pallidum* particle agglutination (TPPA; Fujirebio Inc, Tokyo, Japan) [18]. Urine samples were tested for NG/CT using DNA amplification methods (Amplicor NG/CT Test, Roche Diagnostics, Branchburg, New Jersey, USA). Positive samples were confirmed with the LCx™ NG*/*CT Assay (Abbott Laboratories, Chicago, Illinois, USA) in 1998 and the BD ProbeTec CT/NG (Becton Dickinson & Co., Cockeysville, Maryland, USA) in 2008. Finally, for TV diagnosis in women, a culture using the InPouch™ TV (Biomed Diagnostics, San José, California, USA) was used during both rounds.

**Figure 2:**
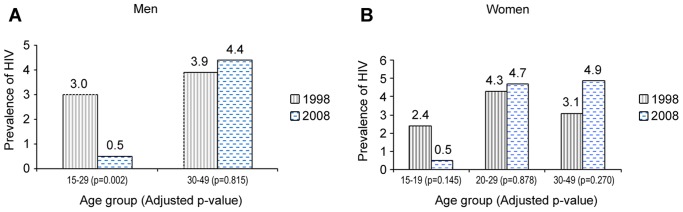
Multivariate comparison by age group of HIV prevalence between 1998 and 2008 among men and women, general population of Cotonou, Benin. P-values are from Wald chi-square test from multivariate logistic models (taking into account the cluster affect and adjusting for marital status). There is a significant decline in HIV prevalence in men aged 15–29 years and a non-significant decrease among women aged 15–19 years. Irrespective of gender, there is a non-significant increase in HIV prevalence in all the other age groups. There were too few HIV-infected men aged 15–19 years in both studies (zero in 1998 and only one in 2008) to further break down the 15–29 age group among men.

### Statistical Analyses

Data were double-entered with EPI-INFO (CDC, Atlanta, Georgia, USA) and analysed with SAS 9.2 (SAS Institute, Inc., Cary, North Carolina). Descriptive comparisons of socio-demographic, sexual behaviour and laboratory variables using the Roa-Scott Chi-square test (proportions) or the t-test (means), taking into account the cluster effect, were performed to compare participants in both studies. Changes in HIV/STI prevalence were described after stratification for age and gender. Multivariate logistic regression models (taking into account the cluster effect and adjusting for socio-demographic variables) were performed for the analyses of HIV/STI trends. Finally, using identical multivariate logistic regression and the pooled data (1998 and 2008), we assessed the association between HIV prevalence and the main socio-demographic and behavioural factors collected in both study rounds for all respondents and then separately for men and women (three different models are presented). We also systematically considered the statistical interaction between the study round and factors significantly associated with HIV. Where this interaction is significant, the association between the risk factor and HIV is presented separately for each round. Adjusted odds ratios with their 95% confidence intervals were computed for all the variables kept in the model (all factors statistically significant at a 0.05 two-tailed alpha threshold in at least one of the models were kept in the three final models) and a test for trend was carried out for variables with multiple ordered categories.

**Table 2:** Comparison of sociodemographic characteristics and sexual behaviour between 1998 and 2008 among women and men aged 15–49 years, general population of Cotonou, Benin.

Demographic characteristics & sexual behaviour	1998 (N = 2112)	2008 (N = 2507)	p-value†
**Age [mean ± Standard error]**			
*Women*	28.1 (±0.3)	28.6 (±0.3)	0.240
*Men*	27.6 (±0.3)	28.2 (±0.3)	0.193
*Overall*	27.9 (±0.2)	28.4 (±0.2)	0.084
**Marital status (%)**			
*Women*			0.0003
Married	600/1091 (55.0)	833/1340 (62.2)	
Separated-Divorced-Widowed	93/1091 (8.5)	134/1340 (10.0)	
Single	398/1091 (36.5)	373/1340 (27.8)	
*Men*			0.158
Married	415/1018 (40.8)	482/1154 (41.8)	
Separated-Divorced-Widowed	21/1018 (2.1)	40/1154 (3.5)	
Single	582/1018 (57.2)	632/1154 (54.8)	
**Education level (%)**			
*Women*			<0.0001
None	409/1091 (37.5)	408/1343 (30.4)	
Primary*	640/1091 (58.7)	661/1343 (49.2)	
Secondary	34/1091 (3.1)	211/1343 (15.7)	
University	8/1091 (0.7)	63/1343 (4.7)	
*Men*			<0.0001
None	90/1018 (8.8)	100/1158 (8.6)	
Primary	754/1018 (74.1)	348/1158 (30.1)	
Secondary	116/1018 (11.4)	516/1158 (44.6)	
University	58/1018 (5.7)	194/1158 (16.8)	
**Age at first sex [mean ± SE^‡^]**			
*Women*	17.9 (±0.1)	17.8 (±0.1)	0.553
*Men*	17.8 (±0.1)	17.8 (±0.1)	0.789
*Overall*	17.9 (±0.1)	17.8 (±0.1)	0.577
**Age at first marriage [mean ± SE^‡^]**			
*Women*	20.5 (±0.2)	21.8 (±0.2)	<0.0001
*Men*	25.8 (±0.3)	25.2 (±0.2)	0.099
*Overall*	22.5 (±0.2)	23.1 (±0.2)	0.049
**Sexually active [n/N (%)]**			
*Women*	950/1091 (87.1)	1220/1344 (90.8)	0.007
*Men*	892/1019 (87.5)	1032/1159 (89.0)	0.342
*Overall*	1842/2110 (87.2)	2252/2503 (90.0)	0.008
**Ever used condom [n/N (%)]**			
*Women*	108/950 (11.4)	428/1220 (35.1)	<0.0001
*Men*	261/892 (29.3)	630/1032 (61.0)	<0.0001
*Overall*	369/1842 (20.0)	1058/2252 (47.0)	<0.0001
**Paid or being paid for sex during the last year [n/N (%)]**			
*Women*	11/1093 (1.0)	18/1341 (1.3)	0.453
*Men*	67/1019 (6.6)	146/1159 (12.6)	<.0001
*Overall*	78/2112 (3.7)	164/2500 (6.6)	<.0001
**Number of sexual partners during lifetime [mean ± SE^‡^]**			
*Women*	2.3 (±0.2)	2.4 (±0.1)	0.481
*Men*	7.2 (±0.5)	7.0 (±0.4)	0.672
*Overall*	4.7 (±0.3)	4.5 (±0.2)	0.609

† p-value of Rao-Scott Chi-Square or t-test taking into account the cluster effect, ^‡^standard error, *completed or not.

### Ethical Considerations

Verbal informed consent was obtained from all the selected participants (separately for the interview and for each biological specimen). Verbal consent was preferred to written consent to ensure full anonymity of the participants on all documents related to the study in the context of HIV-related stigma. In 1998, the verbal consent was documented on the consent forms by the signature of the interviewer, whereas a witness, completely independent of the study staff, confirmed in writing the verbal consent of the participant in 2008. Signed consent forms were kept along with the questionnaires and a copy was given to each participant. Both studies, including the consent procedures and forms, were approved by an ad hoc ethics committee convened by the Ministry of Health in Benin and the Institutional Review Board (IRB) of the Institute of Tropical Medicine (Antwerp, Belgium). Additional approval was obtained from the ethics committee of the London School of Hygiene and Tropical Medicine in 1998, and from the IRBs of the University Teaching Hospital in Antwerp and the Centre hospitalier *affilié* universitaire de Québec in 2008.

## Results

Overall, 2,112 men and women were recruited in 1998 and 2,507 in 2008. The majority of these subjects were female (52% in 1998 and 54% in 2008). In each study, 96.1% of the women agreed to be interviewed whereas 89.3% (1998) and 87.4% (2008) agreed to provide a venous blood sample. Among men 94.5% (1998) and 91.2% (2008) accepted the interview while 85.9% (1998) and 80.2% (2008) provided a venous blood sample. In addition, 0.2% of women and men provided a DBS sample in 2008. Urine samples were provided by 85.7% (1998) and 88.4% (2008) of the women and by 83.0% (1998) and 81.8% (2008) of the men. In 2008, 1.4% of the women and 2.3% of the men were tested for HIV on urine samples, with resulting proportions of 89.0% and 82.7% providing at least one biological sample (and thus tested for HIV), respectively. The comparison of sexual behaviour and socio-demographic characteristics between subjects who were tested for HIV and those who did not showed a higher proportion of subjects with no formal education among men not tested for HIV: this difference was similar in both rounds (17.2% versus 8.0% in 1998, p = 0.001; 18.8% versus 7.6% in 2008, p<0.0001). Furthermore, in 1998, there was a higher proportion of married subjects among men aged <30 years not tested for HIV compared to those who were tested (34.0% versus 13.8%, p = 0.004), but this difference was not significant in 2008 (19.0% versus 17.7%, p = 0.797) and for older men in either round (80.0% versus 84.4% in 1998, p = 0.388; 78.4% versus 77.9% in 2008, p = 0.938). Regarding the women, no significant difference between tested and untested subjects was observed in either round (data not shown).

**Table 3:** Risk factors for HIV infection using pooled data from the 1998 and 2008 surveys in the general population of Cotonou, Benin.

Factor	HIV prevalence n/N (%)	Adjusted odds ratio (95% CI)&	p-value
**All subjects**			
*Study round*			
Round 1	66/1939 (3.4)	1	–
Round 2	71/2288 (3.1)	0.95 (0.61–1.49)	0.8342
*Age group*			
15–29 years	70/2610 (2.7)	1	–
30 years +	67/1617 (4.1)	1.00 (0.64–1.56)	0.9985
*Educational level*			0.0002†
None	45/899 (5.0)	1	
Primary*	74/2227 (3.3)	0.72 (0.51–1.00)	
Secondary or University**	18/1092 (1.7)	0.38 (0.23–0.65)	
*Marital status*			
Single	33/1851 (1.8)	1	–
Currently married	84/2104 (4.0)	1.76 (1.05–2.95)	0.0312
Divorced/Separated/Widowed	19/261 (7.3)	2.99 (1.56–5.73)	0.0010
*Number of lifetime partners*			
<5	85/3092 (2.8)	1	–
≥5	51/1127 (4.5)	2.12 (1.31–3.44)	0.0023
**Women**			
*Study round*			
Round 1	35/1013 (3.5)	1	–
Round 2	50/1239 (4.0)	1.23 (0.72–2.10)	0.4413
*Age group*			
15–29 years	49/1377 (3.6)	1	–
30 years +	36/875 (4.1)	0.91 (0.54–1.51)	0.7006
*Educational level*			0.0080†
None	38/745 (5.1)	1	
Primary*	41/1210 (3.4)	0.64 (0.41–1.02)	
Secondary or University**	6/290 (2.1)	0.36 (0.15–0.88)	
*Marital status*			
Single	21/725 (2.9)	1	–
Currently married	48/1310 (3.7)	1.11 (0.58–2.11)	0.7629
Divorced/Separated/Widowed	16/210 (7.6)	2.17 (1.01–4.69)	0.0495
*Number of lifetime partners*			
<5	67/2053 (3.3)	1	–
≥5	17/193 (8.8)	2.70 (1.47–4.95)	0.0014
**Men**			
*Age effect in each study round* 1			
Study round 1998			
15–29 years	18/593 (3.0)	1	–
30 years +	13/333 (3.9)	0.48 (0.19–1.22)	0.1236
Study round 2008			
15–29 years	3/640 (0.5)	1	–
30 years +	18/409 (4.4)	5.71 (1.21–26.92)	0.0277
*Educational level*			0.0524†
None	7/154 (4.6)	1	
Primary*	33/1017 (3.2)	0.69 (0.31–1.56)	
Secondary or University**	12/802 (1.5)	0.45 (0.20–1.05)	
*Marital Status*			
Single	12/1126 (1.1)	1	–
Currently married	36/794 (4.5)	4.26 (1.93–9.39)	0.0003
Divorced/Separated/Widowed	3/51 (5.9)	4.76 (1.27–17.92)	0.0210
*Number of lifetime partners*			
<5	18/1039 (1.7)	1	–
≥5	34/934 (3.6)	1.29 (0.68–2.45)	0.4411

1 The p-value for the interaction term between age and study round among men was 0.0005 (Llikelihood Ratio Test**)**;

& 95% confidence interval from multivariate logistic regression taking into account the cluster effect and additionally adjusted for sex in analyses of all subjects;

† p-value for trend in HIV prevalence across increasing educational levels, from logistic regression model adjusted for study round, age, marital status (and the interaction term or the sex when applicable), and taking into account the cluster effect (using educational level as a continuous variable);

* completed or not;

** at least some level of secondary education.

The overall HIV prevalence was 3.4% in 1998 and 3.1% in 2008, but there were considerable differences by sex and age. There was a significant decline in HIV among men (Figure 1 and Table 1), that was more pronounced among those aged 15–29 years (Figure 2), with a six-fold decrease, from 3.0 to 0.5% (p = 0.002). There was also a decline in women aged 15–19 years, but this was not significant (Figure 2). In the older age groups there was an increase in HIV prevalence which was more pronounced in women aged 30–49 years. Figure 1 also shows the prevalence of other STIs in the two studies. Overall, the prevalence of syphilis decreased significantly (p = 0.002, Figure 1C). The decrease in syphilis was significant among women (p = 0.013, Figure 1A) and borderline significant among men (0.097, Figure 1B). Among men, there was a significant decrease in gonorrhea and a significant increase in the prevalence of HSV-2 (p = 0.0001, Figure 1B). There was also an overall increase in HSV-2 prevalence (p = 0.008, Figure 1C). The changes in the prevalence of the various STIs observed between the study rounds remained significant in the multivariate analysis (Table 1).

Table 2 shows the changes in sociodemographic and behavioural factors between the two study rounds. Nearly 90% of the participants were sexually active and this proportion increased between 1998 and 2008 only in women (p = 0.007). The proportion of men and women who reported ever having used condoms increased significantly over time, and it was higher in men (61%) than in women (35%) in 2008. The proportion of participants who reported commercial sex during the last year increased significantly only in men. Irrespective of gender, the proportion of subjects having attained a secondary educational level or more increased significantly over time: from 3.8% to 20.4% among women (p<0.0001) and from 17.1% to 61.3% among men (p<0.0001). Overall, men’s educational level was higher than that of women in both surveys.

In the multivariate analyses of risk factors for HIV infection, there was a significant decrease in HIV prevalence across increasing educational levels in women and all subjects combined, whereas this decrease was borderline significant in men (Table 3). Divorced separated and widowed men and women were more likely to be HIV-positive. Married men also had a higher HIV prevalence than single ones, whereas the prevalence was similar in single and married women. There was a significant four-fold higher HIV prevalence among women who reported having had five lifetime sexual partners or more. In the men, the difference was not significant.

In men, the interaction term between age and study round was statistically significant (p = 0.0005). In 2008, there was a five-fold higher HIV prevalence among men aged 30–49 years compared to younger ones (Table 3), whereas age was not a significantly associated with HIV in 1998. For women as well as for all subjects, there was no significant statistical interaction between age and study round (data not shown). Finally, no other significant interaction was found between any of the other independent variables and study round.

The variable “ever used condom” was not significantly associated with HIV prevalence, although there was a tendency towards a protective effect among men (HIV prevalence of 2.9% among men reporting having ever used a condom vs. 4.2% among the other ones, p = 0.2309).

## Discussion

The major finding of this study is the significant decline in HIV prevalence among young men aged 15–29 years from 1998 to 2008. Among women aged 15–19 years, there was a non-significant decline. We did not assess HIV prevalence among men aged 15–19 years as we did for women, since there were no HIV-infected men in this age group in 1998, and only one in 2008. Our findings are comparable to those of a study conducted in Uganda in the 1990s which showed a significant decrease in HIV prevalence in 13–24 year-old young men and a borderline decline among women aged 13–19 years [23]. A significant decline among men only was also observed more recently in repeated national population-based surveys among young people aged 15–24 years in South Africa and Tanzania [3], [24].

In our study, there was a significant decrease in gonorrhea in men, the latter being considered as a good marker for evaluating recent sexual behavioural risk for HIV transmission [25]. Even if the protective effect of having ever used a condom on HIV risk was not statistically significant, the significant increase in condom use between 1998 and 2008 has certainly played an important role in the gonorrhea and HIV prevalence decline. The absence of a significant association between having ever used a condom and HIV prevalence could be related to the cross-sectional design of the surveys that raised temporality issues. Indeed, people with high-risk behaviour may start using condoms after becoming aware of their risk of being HIV-positive [26]. Overall, the decline in HIV prevalence among men was not associated with noteworthy changes in sexual behaviour. In 2008, compared to men aged >30 years, HIV prevalence was significantly lower in the younger age group. The decrease in HIV prevalence in young men happened while an intensive intervention targeting the sex work milieu was ongoing. As shown by mathematical modelling using data from Cotonou, reinforcement of preventive interventions targeted at the sex work milieu can result in a significant decrease in both the incidence and prevalence of HIV/STI in the general population [16]. The intervention implemented in Cotonou from 1992 onwards was strengthened between 2001 and 2006. During this period, 71% of the clients of FSWs were aged 15–29 years, and it was estimated that 30% of the adult males used commercial sex services during the previous year [12], [27]. Thus, the intervention may have reached more young men aged 15–29 years than older ones. Overall, the men of the general population may have been more extensively covered by these preventive interventions than the women. Indeed, a higher proportion of men reported condom use than women in both rounds. There was also an increase in the proportion of men with at least secondary education between the two study rounds. Our results indicating a significant decreasing trend in HIV prevalence across increasing educational levels are consistent with those of previous studies in sub-Saharan Africa [28], [29]. Educational improvement, which is a distal determinant of HIV, is important for a greater impact of preventive interventions on HIV risk reduction and is therefore part of combination prevention responses [30]. In our analyses, we thus made the choice to assess the difference in HIV and STI prevalence over time without adjusting for educational level. We indeed assumed that this factor could not be considered as a confounder, but rather as an intermediate factor that could have led, at least in part, to the observed changes over time, especially given the huge increase in educational level we observed between 1998 and 2008.

The national programme for access to ART began in Benin only in 2002 and the proportion of treated subjects among those eligible for ART increased from almost none in 1998 to 72% in 2008 [31]. Thus, the upward trend in HIV prevalence among the oldest subjects is likely related, at least in part, to better survival rates among those receiving ART. It has been noted that HIV prevalence in young people can indirectly approximate HIV incidence among them [24], [32]. Accordingly, the proportion of HIV-infected subjects with an advanced infection is likely to be higher in older people. Thus, we can assume that in settings (like Cotonou) where, during the study period, ART was initiated in patients with advanced infection (CD4<200/mm^3^ or WHO clinical stage 3 with CD4<350/mm^3^ or WHO stage 4 regardless of CD4 cell count) [33], older people were more likely to be treated. Because of such reasons, an increase in HIV prevalence of 3.6% in women and of 2.5% in men (30–34 year-olds) was observed in South Africa in 2008 as a result of the impact of successful antiretroviral therapy on improved survival [34].

Our data suggest an increase in the proportion of men who paid for sex during the last year. Other studies suggest that the proportion of men having sexual intercourse with FSWs may have been under-reported in 1998 [35]. The observed increase could thus be partially due to a decrease in the social desirability bias for reporting this behaviour [36]. Nevertheless, risky sexual behaviour is still prevalent as shown by the 2008 data.

In both men and women, being widowed/divorced/separated was significantly associated with higher HIV prevalence. This has also been observed in many other studies carried out in the general population in developing countries [12], [26]. Indeed, separated, divorced and widowed women tend to be socially and culturally isolated and are likely to be pressurised to meet the needs of their family. Such women might be forced into situations which make them vulnerable for sexual exploitation indirectly rendering them vulnerable to HIV. On the other hand, their male counterparts may get involved in risky sex to fulfill their sexual needs, as they do not have a stable partner anymore. Finally, couple’s dissolution and widowhood could also be related to HIV issues in one of the partners (marital conflict because of HIV status, AIDS-related death). While higher HIV prevalence among women was associated with having more than five lifetime sexual partners, this association was not significant in men. This result is similar to that of a population-based study in 2005 in Côte d’Ivoire where HIV prevalence was four-fold higher among women with six to ten lifetime partners compared to women with only one, whereas among men, no such association was observed [5]. Men have more often a high number of sexual partners than women and there is subsequently more heterogeneity of risk among them than among the few women having a high number of partners, making it more difficult to identify high number of sexual partners as a risk factor for HIV among men.

The decline in NG prevalence among men could be related to the positive impact of FSW preventive interventions as reported in several studies [14], [37]. In addition to the potential impact of FSW interventions and increased educational level, the overall decline in syphilis could be related in part to systematic screening and treatment of this infection during antenatal visits [38], and also during premarital check-ups which are now an encouraged practice in Benin.

The observed increase in HSV-2 prevalence between the two study rounds could be related to the fact that, during sexual intercourse, condom use protects less against HSV-2 acquisition than against HIV. In previous studies, consistent condom use has been found to be associated with 30% and 80–96% reduction in incidence of HSV-2 and HIV, respectively [39]–[41]. Continuous risk taking combined with differences in the protective effectiveness of condom use could explain why HSV-2 prevalence increased in 2008 among men in general and in young men in particular, in contrast to the decrease observed in HIV and gonorrhoea prevalence. Moreover, the decrease in CD4 cell count over time in HIV infected subjects may increase the risk of HSV-2 acquisition [42] and more frequent herpetic ulcers in HIV/HSV-2 co-infected subjects will increase the likelihood of onwards HSV-2 transmission [43]. It is therefore to be expected that HSV-2 prevalence increases as the HIV epidemic matures, since an increasing proportion of HIV-infected subjects is at an advanced stage of the disease [44]. Finally, since the Kalon test has been shown to have a somewhat higher sensitivity than that of the Gull, but a similar specificity, and given the previously-observed wide variability in performance between HSV-2 specific antibody detection tests [22], [45], we cannot exclude the potential impact of a change in testing method on the observed trend.

There are some methodological issues that could have impacted on the validity of our results. Firstly, two different kits were used for HIV confirmation (Capillus HIV1/2 in 1998 and Genie II® in 2008); however the reported performances for both are similar [46]. In addition, the HIV screening test used on urine samples of a few subjects has a reported sensitivity of 100% and specificity of 98.7%; however the use of Western Blot confirmation enables the elimination of false positive results [47]. It is therefore unlikely that differences in the tests used between the two rounds are responsible for the significant decline in HIV prevalence observed in young men. Different tests for the confirmation of NG and CT were also used between 1998 and 2008, but their reported performances were similar and were then unable to artificially induce the observed trends [48]. Secondly, we did not standardise our comparisons on the basis of the differences in age distribution between the two rounds. However, given a similar age distribution in both rounds, this is very unlikely to have biased our analyses. Thirdly, differential response rates in the two surveys could have biased our analyses, but fortunately, we had high and comparable participation rates in both rounds (for HIV testing, 85.9% in 1998 and 82.7% in 2008 among men, and corresponding figures of respectively 89.3% and 89.0% among women). When comparing subjects who gave a biological sample for HIV testing to those who did not, there were few differences among men and no significant ones among women. Non-response therefore seems unlikely to have resulted in substantial overestimation of the decline in HIV prevalence.

Finally, the information collected in 1998 was insufficient to estimate the weights of the sampling units for analyses. However, in both 1998 and 2008, the primary units were sampled based on a “probability proportional to size” method, and approximately the same number of households were selected by segmentation of each cluster. The samples can thus be considered as self-weighting samples, unless response rates vary greatly between clusters or study rounds. Since the response rates were high and similar between rounds, it is unlikely that using unweighted logistic regression could affect the validity of our results. We also adjusted our analyses for the cluster effect to take into account a potential type I error due to dependency between observations. Overall, we thus conclude that our results are valid.

### Conclusions

This is the first study that reports a significant decline in HIV prevalence among young men in a sub-Saharan Africa country where overall prevalence has never reached 5%. The decline occurred when a preventive intervention targeting the sex work milieu was ongoing and while the educational level was increasing.
